# Reducing Substances in the Urines of Alcoholics

**Published:** 1908-05-16

**Authors:** 


### REDUCING SUBSTANCES IN THE URINES OF ALCOHOLICS.

It constantly happens in the casualty department of any large hospital that patients are brought in comatose or semi-comatose, and the question of whether the unconsciousness is due solely to alcoholism, or to something else as well has to be solved as soon as possible. Similar cases come with almost greater frequency under the notice of police divisional surgeons, and even in private practice occur from time to time.

It is important, therefore, to know that the urine of a patient suffering from acute alcoholism may reduce Fehling's solution with such readiness that it is possible to mistake the condition for diabetes mellitus. This is more particularly likely to occur if diacetic acid and acetone are regarded as distinctive of diabetes, for both these substances may occur in the urine in small but definite quantities along with the reducing substance in these cases.

Investigations have been made into the nature of the reducing substance, and it has been proved to be not glucose but a conjugated glycuronic acid. Its presence is temporary only, and it may be entirely absent from the patient's urine upon the day following the alcoholic bout, or at longest upon the day after that.

The distinction between this reducing substance and glucose is easy if the fermentation test is employed. Urine containing glucose readily ferments, whereas urine which contains the glycuronate does not do so at all.

The phenylhydrazine test will also serve to distinguish the two if one is familiar with the various crystals; but it needs to be borne in mind that glycuronates will sometimes yield yellow needle-shaped crystals with phenylhydrazine, and that although these are different in form from those of phenylglucosazone, they may be mistaken for the latter if one is not in the habit of seeing each kind of crystal from time to time.

Another point of distinction is that the glycuronate is lævo-rotatory, whereas glucose is dextro-rotatory. This test, however, is scarcely applicable except in a special laboratory. Since the reduction of alcoholic urine is transient, a simple and rapid test is needed to distinguish between alcoholic and diabetic coma.

